# Reconstruction of severe burn contractures of the upper lip in males using a pedicled superficial temporal artery hair-bearing flap. Two case reports

**DOI:** 10.1016/j.ijscr.2020.06.012

**Published:** 2020-06-12

**Authors:** Mohammad M. Al-Qattan, Musa H. AlMutairi

**Affiliations:** a Department of Surgery, King Saud University, P.O. Box 18097, Riyadh, 11415, Saudi Arabia; b Department of Plastic Surgery at Prince Sultan Military Medical City, Riyadh, Saudi Arabia

**Keywords:** Reconstruction, Burn contracture, Upper lip, Males, Pedicled superficial temporal artery hair-bearing flap

## Abstract

**Introduction:**

Severe burn contractures of the upper lip are usually treated either with full thickness skin grafts or flaps. Most authors recommend the free radial forearm flap as the flap of choice. In males, another free flap option is the use of the superficial temporal artery hair-bearing free flap.

**Case reports:**

In this report, we demonstrate that the superficial temporal artery hair-bearing flap may be used as a pedicle flap (rather than a free flap) for the reconstruction of severe upper lip burn contractures in males. We show our method of flap delay and design to ensure that the distal part of the flap will survive; and this will be demonstrated in two case reports.

**Discussion:**

The pedicled superficial temporal artery flap is much simpler to execute than free flaps.

**Conclusions:**

Severe post burn contractures of the upper lip in males may be reconstructed using the pedicled superficial temporal artery hair-bearing flap. In order for the flap to reach the contralateral side of the lip, it will have to cross the midline in the scalp. We demonstrate our method of flap delay and design to ensure flap safety and viability. We also show that concurrent burn deformities in close proximity of the upper lip should be treated prior to upper lip reconstruction. Finally, we stress on the principle of reconstructing the entire skin of the upper lip as one aesthetic unit.

## Highlights

• The use of the superficial temporal artery free flap has been described for upper lip burn contractures.
• However, the pedicled superficial temporal artery flap is much simpler to execute than free flaps.
• The main concern with the use of the pedicled superficial temporal artery flap in these cases is regarding the viability of the distal end of the flap.
• We demonstrate this in two case reports.

## 1 Introduction

The choice of reconstruction of burn contractures of the upper lip depends on the severity of the contracture and associated deformity. Minor contractures or deformities are treated using various techniques of cheiloplasty [1]. Severe contractures are treated either with full thickness skin grafts or flaps. Flap options include pedicled nasolabial flaps or free flaps [2]. Most authors recommend the free radial forearm flap as the flap of choice [2,3]; although other free flaps (such as those harvested from the back) have been described [4]. In males, another free flap option is the use of the superficial temporal artery hair-bearing free flap [5].

In this report, we demonstrate that the superficial temporal artery hair-bearing flap may be used as a pedicle flap (rather than a free flap) for the reconstruction of severe upper lip burn contractures in males. We show our method of flap delay and design to ensure that the distal part of the flap will survive and reach the contralateral side of the lip under the no tension; and this will be demonstrated in two case reports. This work has reported in line with the SCARE criteria [6].

## 2 Case reports

### 2.1 Case #1

**Fig. 1:**
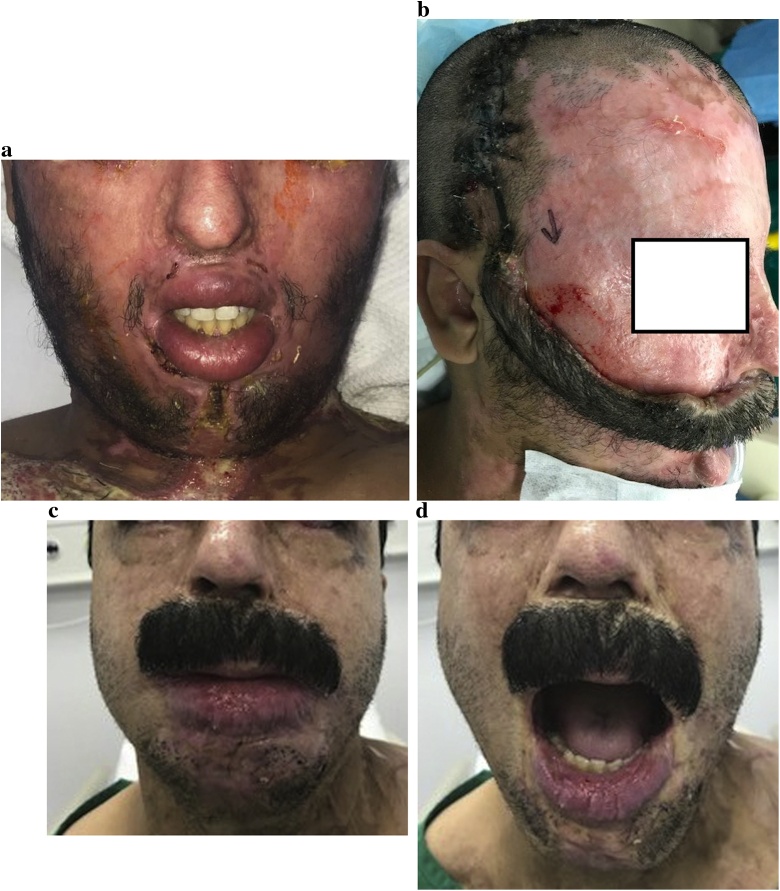
Case #1. A) Pre-operative appearance. Note the tight oral commissures, B) flap transposition. Note that the undersurface of the flap pedicle is covered with a skin graft, C) The cosmetic result at one year is satisfactory, D) The functional outcome is excellent with adequate release of the oral commissures.

A 38-year old male sustained a chemical burn to the face from an assault. The burn was treated conservatively at a local hospital. The case was transferred to us two months later. There was severe upper lip burn contracture and tight oral commissures (Fig. 1a). Bilateral commissuroplasty and insertion of the expander in the scalp were done in the first stage. Removal of the expander and flap transposition was done in the second stage (Fig. 1b). The flap was divided in 3 weeks in the third and final stage. At final follow-up one year later, the cosmetic and functional outcomes were satisfactory and the patient was satisfied with the results (Fig. 1c, d).

### 2.2 Case #2

**Fig. 2:**
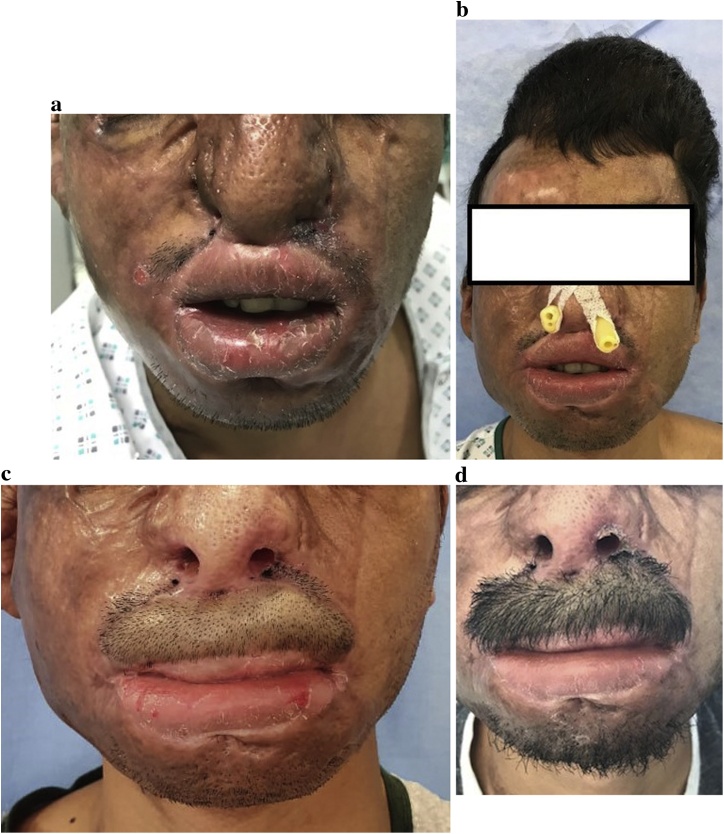
Case #2. A) Pre-operative appearance. Note the concurrent tight oral commissures and the severe bilateral nostril stenosis. Also note that the skin deficiency in the upper lip is more severe in the central part of the lip, B) Appearance after the first stage during which 3 procedures were done: commissuroplasty, W-plasty/stenting of the nostrils, and the insertion of the tissue expander in scalp. Note that the expander crosses the mid line in the scalp, C) Appearance at 9 months. The moustache was intentionally shaved to show that the reconstruction was done as one aesthetic unit. Some of the native skin from the lateral aspects of the lip was excised to achieve this goal (compare to Fig. 2a), D) Appearance with scalp hair growth within the flap. The patient is taught to do regular trimming of the hair.

A 25-year old male sustained a chemical burn to the face at work. The burn was treated conservatively at a local hospital. The case was transferred to us 5 months later. There was an upper lip contracture which is more severe in the central part of the lip. There was also bilateral severe nostril stenosis and bilateral tightness of the oral commissures (Fig. 2a). The first stage included bilateral commissuroplasty, bilateral nostril stenosis correction, and the insertion of the tissue expander in the scalp (Fig. 2b). In the second stage, the upper lip contracture was released. Some of the native upper lip skin was excised in order to provide the reconstruction in the form of one aesthetic unit. The flap was divided in 3 weeks in the third and final stage. At final follow-up 9 months later, the cosmetic and functional outcomes were satisfactory and the patient was satisfied with the result (Fig. 2c, d).

### 2.3 Method of flap delay and design

The head is shaved. The superficial temporal artery and its parietal branch are identified and marked on the scalp with the help of a doppler. The pivot point in the pre-auricular area is marked and a piece of gauze is used (fixed at the pivot point) to simulate flap length and rotation to ensure that the flap will reach the contralateral side of the lip under no tension. The flap design is also made so that the parietal branch is within the center of the flap. In order for the flap to reach the contralateral side of the lip, it will have to cross the midline in the scalp. Hence, the parietal branch of the contralateral superficial temporal artery flap is also marked. The right and left parietal branches are known to anastomose with each other across the mid line in the scalp [7]. The design of the flap across the mid line in the scalp should include this anastomotic area (also known as the “choke” zone) between the two patietal branches. Once the flap is marked on the scalp, an incision is made 2 cm distal to the most-distal part of the flap; dividing the contralateral parietal branch. A rectangular tissue expander is inserted under the flap and its base. Both the incision at the distal part of the flap and the expansion process will act as a “delay” to boost the blood supply of the distal part of the flap at the “choke” zone across the midline [8,9]. Once the expansion process is completed, the flap is raised and transferred to the upper lip defect following the release of the contracture. The flap is inset and sutured into the defect, making sure that the direction of hair is downwards. The undersurface of the flap pedicle is covered with a thin split-thickness skin graft. The flap is divided at 3 weeks.

## 3 Discussion

Defects involving the hemi-upper lip in males are easily reconstructed with an ipsilateral pedicled superficial temporal artery hair-bearing flap [10]. In these cases, there is no need for flap delay because the entire flap design is within the ipsilateral scalp without crossing the midline. If the defect involves the entire skin of the upper lip, authors recommend the use of full-thickness skin grafts or free flaps [[2], [3], [4], [5]]. Full-thickness grafts may be effective to correct the burn contracture, but the aesthetic outcome is poor. In males, the use of the superficial temporal hair-bearing free flap has been described as the flap of choice because of the simultaneous reconstruction of the moustache hides the burn scars [5]. However, the pedicled superficial temporal artery flap is much simpler to execute than free flaps. The main concern with the use of the pedicled superficial temporal artery flap in these cases is regarding the viability of the distal end of the flap.

The concept of angiosomes and choke zones has been introduced by Taylor since the 1980’s [11]. The angiosomes of the two parietal branches of the superficial temporal arteries join across the scalp in the midline via a choke zone of small terminal blood vessels [8,9]. The delay process will result in the dilatation of these small vessels in the choke zone; and our case reports demonstrate the safety of the reconstructive procedure using the method described earlier.

The other two important messages from our report is regarding the correction of concurrent deformities and the reconstruction of the upper lip as one aesthetic unit. It is important to note that most of these burn patients will have some residual moustache hair in the upper lip and the skin deficiency may not be equal across the lip. In order to respect the aesthetic units of the face [12], the entire upper lip skin has to be reconstructed as one unit. This has to be implicated even if one has to excise some of the native skin of the upper lip. Another point that has to be taken in consideration is the correction of concurrent burn deformities in close proximity of the upper lip. The most two common deformities seen in these patients are: tightness of the oral commissures and nostril stenosis. Commissuroplasty and correction of nostril stenosis should be done prior to upper lip reconstruction. For nostril stenosis we use the W-plasty technique followed by prolonged stenting of the nostrils [13].

## 4 Conclusions

Severe post burn contractures of the upper lip in males may be reconstructed using the pedicled superficial temporal artery hair-bearing flap. In order for the flap to reach the contralateral side of the lip, it will have to cross the midline in the scalp. We demonstrate our method of flap delay and design to ensure flap safety and viability. We also show that concurrent burn deformities in close proximity of the upper lip in the oral commissures and nostrils should be treated prior to upper lip reconstruction. Finally, we stress on the principle of reconstructing the entire skin of the upper lip as one aesthetic unit; even if one has to excise some of the native skin of the lip.

## Declaration of Competing Interest

None.

## Sources of funding

None.

## Ethical approval

The study was approved by the research committee, National Hospital (Care), Riyadh, Saudi Arabia.

## Consent

Written informed consent was obtained from both patients for publication of the case reports and accompanying images. A copy of the written consent is available for review by Editor-In-Chief of this journal on request.

## Authors contributions

Both authors contributed significantly and in agreement with the content of the manuscript. Both authors participated in data collection and in writing of the manuscript.

## Registration of research studies

Not relevant here.

## Guarantor

M.M. Al-Qattan.

## Provenance and peer review

Not commissioned, externally peer-reviewed.
